# Phyto-assisted synthesis of zinc oxide nanoparticles for developing antibiofilm surface coatings on central venous catheters

**DOI:** 10.3389/fchem.2023.1138333

**Published:** 2023-03-23

**Authors:** Akshit Malhotra, Suchitra Rajput Chauhan, Mispaur Rahaman, Ritika Tripathi, Manika Khanuja, Ashwini Chauhan

**Affiliations:** ^1^ Department of Microbiology, Tripura University, Suryamaninagar, Tripura, India; ^2^ Invisiobiome, New Delhi, India; ^3^ Centre for Advanced Materials and Devices (CAMD), School of Engineering and Technology, BML Munjal University, Gurgaon, Haryana, India; ^4^ Central Instrumentation Centre, Tripura University, Suryamaninagar, Tripura, India; ^5^ Centre for Nanoscience and Nanotechnology, Jamia Millia Islamia, New Delhi, India

**Keywords:** green synthesis, plant mediated synthesis, zinc oxide nanoparticles (ZnO NPs), nanoparticle coatings, anti-biofilm coatings, device associated infections, anti-microbial resistance, medical devices

## Abstract

Medical devices such as Central Venous Catheters (CVCs), are routinely used in intensive and critical care settings. In the present scenario, incidences of Catheter-Related Blood Stream Infections (CRBSIs) pose a serious challenge. Despite considerable advancements in the antimicrobial therapy and material design of CVCs, clinicians continue to struggle with infection-related complications. These complications are often due colonization of bacteria on the surface of the medical devices, termed as biofilms, leading to infections. Biofilm formation is recognized as a critical virulence trait rendering infections chronic and difficult to treat even with 1,000x, the minimum inhibitory concentration (MIC) of antibiotics. Therefore, non-antibiotic-based solutions that prevent bacterial adhesion on medical devices are warranted. In our study, we report a novel and simple method to synthesize zinc oxide (ZnO) nanoparticles using ethanolic plant extracts of *Eupatorium odoratum*. We investigated its physio-chemical characteristics using Field Emission- Scanning Electron Microscopy and Energy dispersive X-Ray analysis, X-Ray Diffraction (XRD), Photoluminescence Spectroscopy, UV-Visible and Diffuse Reflectance spectroscopy, and Dynamic Light Scattering characterization methods. Hexagonal phase with wurtzite structure was confirmed using XRD with particle size of ∼50 nm. ZnO nanoparticles showed a band gap 3.25 eV. Photoluminescence spectra showed prominent peak corresponding to defects formed in the synthesized ZnO nanoparticles. Clinically relevant bacterial strains, viz., *Proteus aeruginosa* PAO1*, Escherichia coli* MTCC 119 and *Staphylococcus aureus* MTCC 7443 were treated with different concentrations of ZnO NPs. A concentration dependent increase in killing efficacy was observed with 99.99% killing at 500 μg/mL. Further, we coated the commercial CVCs using green synthesized ZnO NPs and evaluated it is *in vitro* antibiofilm efficacy using previously optimized *in situ* continuous flow model. The hydrophilic functionalized interface of CVC prevents biofilm formation by *P. aeruginosa*, *E. coli* and *S. aureus*. Based on our findings, we propose ZnO nanoparticles as a promising non-antibiotic-based preventive solutions to reduce the risk of central venous catheter-associated infections.

## 1 Introduction

Intravascular catheters are indispensable in modern day critical care settings (Smith and Nolan, 2013). Annually, in US alone, implantation of 0.15 billion intravascular catheters has been witnessed (Shah et al., 2013). Intravascular catheters like peripherally inserted catheters, central venous catheters and totally implantable venous access ports (TIVAPs) are implanted in patients for various applications such as renal dialysis (Agarwal et al., 2019), nutritional support (Pittiruti et al., 2009), administration of chemotherapy (Kim et al., 2010) and hemodynamic monitoring (Huygh et al., 2016). Unfortunately, the use of central venous catheters is associated with high infection rates (Marco et al., 2018) besides other complications such as mechanical failure (Copetti and de Monte, 2005) and thrombosis (Lebeaux et al., 2014b). In clinics, ∼5%–8% TIVAPs get contaminated by structurally complex microbial biofilm communities due to microbial adhesion upon their surface (Lebeaux et al., 2014a; Stressmann Franziska et al., 2017). Hence, clinicians are challenged with the combined mortality, morbidity and economic burden associated with the use of central venous catheters (Marco et al., 2018). Despite adoption of effective frontline procedures such as prophylactic and therapeutic antimicrobial lock solutions (Niyyar, 2012), aseptic care bundles (O’Grady et al., 2011) or use of next-generation catheter designs employing anti-fouling materials (Ricardo et al., 2020)) deleterious microbial contamination resulting in central-line associated bloodstream infections (CLABSIs) remains an unmet problem (Chopra et al., 2013). CLABSIs originating from biofilm lifestyle of microbes can be difficult to eradicate due to the multi-factorial recalcitrance of microbial biofilms (David et al., 2014; Singh et al., 2021). A recent health-care-associated infection (HAIs) surveillance network in India found *Klebsiella* spp., *Acinetobacter* spp., *Pseudomonas* spp., and *Escherichia* spp. to be the most important etiological agents for CLABSI infections indicating an alarming predominance of Gram-negative bacteria (Mathur et al., 2022). There is a strong need to develop effective strategies which can control infections in Central Venous Catheters caused by such bacterial pathogens, primarily, to avert the risk of bloodstream infections.

In order to prevent and/or treat CVC-associated bloodstream infections, there are no fool-proof solutions apart from removal of contaminated device which increases cost of patient care and associated trauma (LaBella and Tang, 2012). Clinical practice guidelines indicate administration of antibiotic lock therapy to treat catheter-related infections (Mermel et al., 2009). Several antimicrobial lock solutions like minocycline-EDTA (Ferreira Chacon et al., 2011), ethanol (Wolf et al., 2013) and vancomycin-based lock solutions (Safdar and Maki, 2006) are used to salvage CVCs and also maintain patency. Other than the strategies to treat biofilm associated occlusions in CVCs, clinicians choose insertion of the CVC consisting anti-infective surface modification technologies (Casimero et al., 2020). These surface modifications employ antibiotics like minocycline (Raad et al., 1996), rifampin (Hanna et al., 2004), biocides like polyhexanide (Krikava et al., 2011), silver salts (Corral et al., 2003) or noble metal alloy coating (Björling et al., 2018). Despite significant success in reduction of CLABSI rate by use of surface modified CVCs, there are certain limitations to these coating designs. The drawbacks include the limited release kinetics of antimicrobials (Walder et al., 2002; Wang et al., 2018), emergence of antimicrobial resistance (Sampath et al., 2001), and regulatory and safety issues (Guleri et al., 2012).

ZnO shows high biocompatibility to human organs, recognized as safe and approved by FDA as food additive (Puspasari et al., 2022). ZnO NPs are well known for their anti-quorum sensing (Husain et al., 2020), broad spectrum antimicrobial and antibiofilm activity (Mahamuni-Badiger et al., 2020). ZnO NPs of different morphologies have been utilized as antibacterial coating materials in textile fibers (Verbič et al., 2019), anticorrosion coating for metals (Qing et al., 2015), protection coatings for heritage buildings (Cinteză and Tănase, 2020)and antifouling coatings for dental (Moradpoor et al., 2021) and orthopedic implants (Memarzadeh et al., 2015). Although silver NPs are used to develop antibacterial surfaces of CVCs, reports of limited efficacy (Antonelli et al., 2012) and bacterial resistance against Ag NPs (Stabryla et al., 2021) warrant for better solutions. Urinary catheters were functionalized using ZnO NP based formulations (Ivanova et al., 2021), however, there are no reports on development of antifouling coatings on central venous catheters. In this study, we report a novel and facile-green synthesis of ZnO Nanoparticles (ZnO NPs) using extract of *Eupatorium odoratum*, a traditional medicinal plant used by local tribal population of Tripura (Debbarma et al., 2017). We used ZnO NPs, synthesized using phyto-assisted precipitation method, to develop coatings on luminal and outer surfaces of Totally Implantable Venous Access Port (TIVAP, a type of CVC). Green synthesized ZnO NPs conferred antifouling characteristics to the modified surfaces of TIVAP against *Escherichia coli, Proteus aeruginosa* (Gram-negative) and *Staphylococcus aureus* (Gram-positive) bacterial species using *in vitro* CVC continuous flow model system (Chauhan et al., 2012).

## 2 Material and methods

### 2.1 Collection of plant leaves and preparation of ethanolic extract

The plant leaves of *E. odoratum* were collected, washed with distilled water and subsequently dried at room temperature in dark conditions. After this, the dried leaves were ground to powder and 10 g of powder sample was mixed with 100 mL of ethanol for continuous stirring at 150 rpm for 24 h. In order to remove solid sediments, the mixture was then centrifuged at 10,000 rpm for 10 min. The supernatant was filtered using Whatman filter paper No. 1 and later, concentrated using rotary evaporator to obtain crude extract which was further lyophilized and stored at 4°C.

### 2.2 Synthesis of zinc oxide nanoparticles (ZnO NPs)

Zinc Oxide nanoparticles were prepared using phyto-assisted precipitation (Ranpariya et al., 2021) method using highly concentrated Zn precursor, ZnCl_2_. Briefly, 0.2 M ZnCl_2_ was prepared with total reaction mixture volume as 100 mL. Initially, only 80 mL distilled water was mixed with ZnCl_2_ powder using magnetic stirring at 150 rpm at 70°C. After complete dissolution of ZnCl_2_ crystals, the ethanolic extract of *E. odoratum* (10 mg/mL concentration) was added to the reaction mixture with a total volume of 5 mL and later, pH was adjusted to 6. After 1 h of continuous stirring, the mixture was transferred to centrifuge tubes for centrifugation at 15,000 rpm for 10 min at 4°C. The supernatant was discarded and the pellet was transferred to fresh tube and mixed with 50% ethanol (v/v in distilled water). The centrifugation cycle is repeated twice and the obtained pellet was kept in oven to dry for 12 h at 80°C.

### 2.3 Determining physico-chemical properties of synthesized nanoparticles by different characterization techniques

The crystallographic phase of NPs was confirmed by X-ray diffraction analysis (PANalytical, EMPYREAN). The operating volage and current during XRD were 45 kV and 45 mA, respectively and, the diffraction pattern was recorded across the 2θ range of 20°–80° with Cu Kα source (1.5406 Å). The peaks obtained in XRD analysis was corroborated with JCPDS database to determine the phase formation of ZnO nanoparticles. The surface morphological analysis of ZnO NPs was done by FE-SEM microscopy (FE-SEM, Sigma-300, Carl Zeiss) and elemental composition was investigated by EDAX spectroscopy. In order to find the band gap of synthesized ZnO NPs, diffuse reflectance spectroscopy was done (Lambda-365 UV-Vis Spectrophotometer Perkin Elmer) with the reflectance of light in 200–800 nm range. The photoluminescence emission spectrum was recorded at laser wavelength of 355 nm and 285 µW incident power using Witech Alpha 300 RA system. The hydrodynamic size and zeta potential of NPs was measured using Litesizer 500 (Anton Paar GmbH). ZnO NPs suspension in distilled water was passed through 0.22 µm nylon syringe filter and sonicated for 30 min at RT in ultrasonic cleaner (LMUC-3, Labman Scientific Instruments Pvt. Ltd., India) operating at 40 kHZ.

### 2.4 Development of ZnO NP based coatings on totally implantable venous access ports (TIVAP)

The coating suspension consisted of 0.2% (w/v) HPMC and 5% (w/v) ZnO NPs in 81.25% ethanol. The coating solution was infused inside the lumens of TIVAP (2005ISP, Vygon, Ecouen, France) with the help of syringe and immersed in the coating solution. The tubings of TIVAP were in contact with coating solution for 6 h at 60°C under continuous orbital shaking at 150 rpm. After this, the excess solution was removed by immersing the catheter in 1x PBS, flushing the lumen of TIVAP by 1x PBS and therefore, ensuring no occlusion inside the lumen of TIVAP. The coated TIVAP was dried in oven at 80°C for 16 h. In order to confirm the coating of ZnO NPs on surfaces of TIVAP, FE-SEM and EDAX analysis was done (Sigma-300, Carl Zeiss) to further compare uncoated and coated TIVAP surfaces in terms of surface morphology and elemental composition. The catheters were sterilized as described elsewhere (Chauhan et al., 2014) in absolute ethanol.

### 2.5 Bacterial strains and growth media

*S. aureus* MTCC 7443 was grown in Tryptic Soy Broth (TSB) and Gram- *E. coli* MTCC 119 and *P. aeruginosa* PAO1 (kind gift received from Dr. Mohan C. Joshi, Jamia Milia Islamia, N. Delhi, India) were grown in Luria-Bertani broth (LB) at 37°C. For enumerating bacterial cell viability, serially diluted culture was spotted on sterile TSB agar (*S. aureus*) or LB agar (*E. coli* or *P. aeruginosa*) plates and kept for incubation at 37°C for 12–16 h.

### 2.6 Effect of *Eupatorium odoratum* mediated and chemically synthesized ZnO NPs on exponentially growing bacteria

The efficacy of *E. odoratum* mediated or chemically synthesized (kind gift from Dr. Khanuja, Jamia Milia Islamia, N. Delhi, India) ZnO NPs in inhibiting growth of *S. aureus*, *E. coli* or *P. aeruginosa* was checked by recording OD 600 nm at different time intervals using Shimadzu UV-Vis Spectrophotometer. Each treatment was replicated thrice and each experiment was carried out 3 times. The exponentially (OD600 ≈ 0.3–0.5) grown bacterial culture of *S. aureus*, *E. coli* or *P. aeruginosa* was given treatment of different concentrations of ZnO NPs and allowed to incubate for 24 h at 37°C with continuous shaking at 150 rpm. The untreated wells were kept as controls. After the incubation, the colony forming units were estimated by plating the serial dilutions on LB agar or TSB agar media plates for Gram-negative and Gram-positive bacteria respectively, and incubated at 37°C for 12–16 h (Lebeaux et al., 2014a).

### 2.7 Evaluation of *in vitro* antibiofilm efficacy of surface modified TIVAP using continuous flow system

The antibiofilm efficacy of surface modified TIVAP was evaluated using continuous flow system described previously (Chauhan et al., 2012) with few modifications. Briefly, bacterial biofilms of *S. aureus*, *E. coli* or *P. aeruginosa* were allowed to form on the ZnO NPs coated, HPMC coated and uncoated TIVAP. Under sterile conditions in laminar airflow, the catheters were supplied with fresh media from reservoir bottles. The intraluminal sections of TIVAP were filled with *S. aureus*, *E. coli* or *P. aeruginosa* at a cell density 100 cells/50 µL and allowed to adhere on catheter’s internal surface at 37°C for 3 h. Later, non-adherent bacteria were removed by flushing out 1x PBS for a duration of 10 min followed by continuous supply of media at a speed of 300 μL/min for 48 h. The non-adherent cells and spent media were collected in the discard bottle from the catheters. After 48 h, the biofilm bacterial cells from intraluminal section of TIVAP were harvested by a vigorous process as described previously (Chauhan et al., 2012). The TIVAP catheter surface was wiped using 70% ethanol to remove any contaminants present on the outer lumen of the TIVAP catheter. Under sterile conditions, the lumen of the catheter was cut cross sectionally in small pieces followed by transversal cuts to expose the inner lumen of the catheter. The cut sections were immersed in 1 mL 1xPBS containing tube and vortexed for 1 min. This was followed by sonication for 5 min using water bath ultrasonic cleaner (LMUC-3, Labman Scientific Instruments Pvt. Ltd., India) operating at 40 kHZ followed by vortex mixing for 1 min. Later, the bacterial cells in the 1xPBS suspension were diluted serially and plated on TSB agar (*S. aureus*) or LB agar (*E. coli* or *P. aeruginosa*) for viable cell count estimation.

## 3 Results and discussion

### 3.1 Phyto assisted ZnO NPs show hexagonal phase with wurtzite structure

**FIGURE 1:**
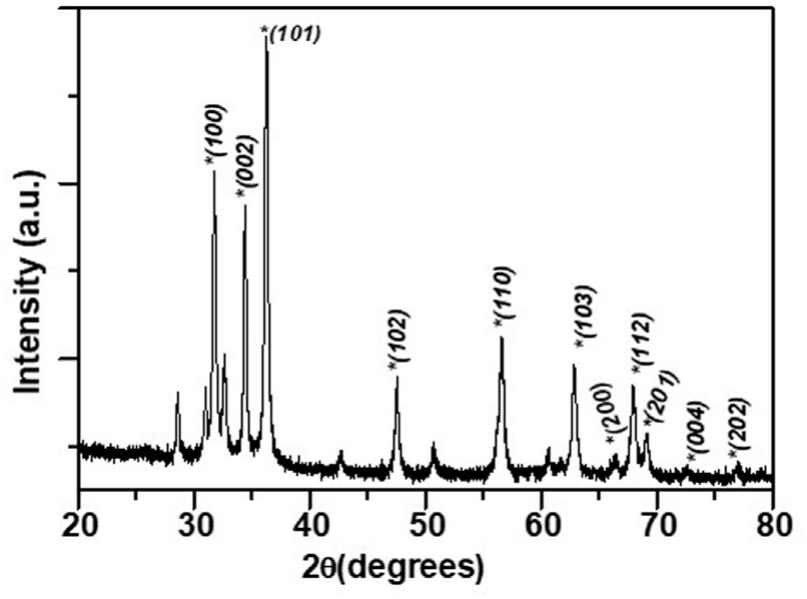
XRD pattern for the synthesized ZnO NPs.

The phase of the ZnO NPs synthesized using ethanolic extracts of *E. odoratum* was identified with the help of X ray diffraction pattern as shown in Figure 1. A series of diffraction peaks due to (100), (002), (101), (102), (110), (103), (200), (112), (201), (004), (202) planes were observed from the synthesized ZnO NPs. A JCPDS file 36-1451 was used to identify the hexagonal phase with wurtzite structure in the synthesized ZnO NPs. Spurious low intensity peaks were also observed specifically between 20° and 35°. These peaks could be due to the intermediate product or impurities. Confirmation of multiphase or impurity was done by EDAX measurements. Only “Zn” ad “O” signals were observed in sample indicating that unidentified peaks in observed XRD pattern corresponds to the intermediate phase only. Similar observation has been reported by Luković Golić et al. (2011), and others (Tokumoto et al., 2002).

Average crystallite size of the ZnO NPs have been determined from the Williamson-Hall plot (W-H plot) (βCosθ versus 4Sinθ) (Jeffery, 1957) after determining the FWHM of the XRD peaks and considering instrumental broadening.

$$\begin{array}{c} \beta_{Total}=\beta_{Size}+\beta_{Strain}=\frac{0.9\lambda }{t\cos\theta }+\frac{4\left(\Delta d\right)\mathrm{Sin}\theta }{dCos\theta }\\ \beta_{Total}\cos\theta =\frac{0.9\lambda }{t}+\frac{4\left(\Delta d\right)\mathrm{Sin}\theta }{d} \end{array}$$ (1)

**FIGURE 2:**
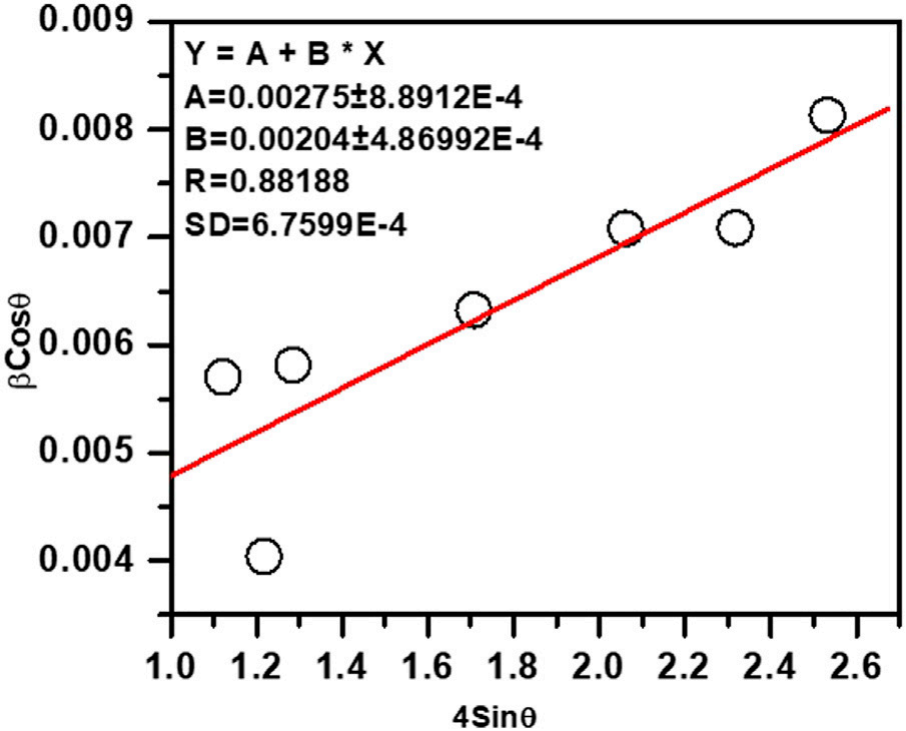
Williamson-Hall plot for the ZnO NPs, particle size was determined from the intercept.

Where, *θ* denotes the Bragg angle, *t* represents the crystal or particle size of ZnO NPs synthesized using ethanolic extracts of *E. odoratum*, *d* represents interplanar lattice spacing, *β*_*Size*_ and *β*_*Strain*_ represent the FWHM contributions pertaining to the size and strain, respectively. *∆d/d* is the measure of strain. FWHM was obtained by fitting individual XRD peak to Lorentzian peak. Figure 2 shows the W-H plot yielding ZnO particle size = 50.4 nm.

The lattice parameters of synthesized ZnO NPs were obtained using an interactive powder diffraction data interpretation and indexing program (Wu, 1989). The indexing program yields lattice parameters by least square fitting to the positions of x ray diffraction peaks in 20–80° range. Lattice parameters a = 3.25500 ± 0 Å and c = 5.21459 ± 0.00091 Å were fitted at figure of merit, F = 33.9 and R = 0.00011. The low value of the R-factor (∼10^−3^) and high value of F > 10 were indicative of the satisfactory estimate of the lattice parameters.

### 3.2 FE-SEM and EDAX spectroscopy analysis

**FIGURE 3:**
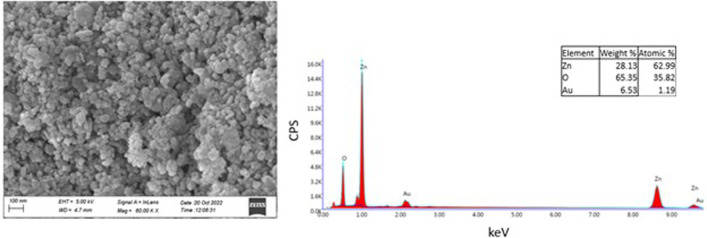
SEM and EDAX Analysis. Field Emission-Scanning Electron Microscopy Images of Zinc Oxide Nanoparticles synthesized using green synthesis showing spherical, hexagonal structures of Zinc Oxide. EDAX spectra reveals presence of Zn and O confirming pure synthesis of Zinc Oxide nanoparticles.

The morphology of ZnO NPs was investigated using FE-SEM microscopy. The spherical and hexagonal morphologies were observed (Figure 3). The SEM micrographs reveal particle aggregation and homogenous morphology distribution in agreement with earlier reports (Chaudhuri and Malodia, 2017; Naseer et al., 2020; Faisal et al., 2021; Iqbal et al., 2021). However, in the previous studies green synthesis procedure has also resulted in irregular crystal growth of ZnO NPs (Ogunyemi et al., 2019; Priyadharshini et al., 2022). The average grain size of *E. odoratum* leaves’ ethanolic extract mediated ZnO NPs was 34 ± 7.98 nm in our study which is in close agreement with XRD findings. As per previous findings, using SEM analysis different size ranges of bio-fabricated ZnO NPs have been reported such as 45–150 nm using cell free extract of *Bacillus megaterium* (Saravanan et al., 2018), 25–87 nm using fruit, seed and pulp extract of *Citrullus colocynthis* (Azizi et al., 2017), 10–20 nm (Doan Thi et al., 2020) using orange fruit peel extract, 30–43 nm using *Withania somnifera* root extract (Prasad et al., 2021), etc. In EDAX spectroscopy, the synthesized nanoparticles showed presence of Zn (65.35 wt%), O (28.13 wt%) and Au (6.53 wt%, due to gold sputter coating prior to SEM microscopy) indicating successful synthesis of ZnO nanoparticles.

### 3.3 UV-visible absorption and diffuse reflectance spectroscopy (DRS) analysis

**FIGURE 4:**
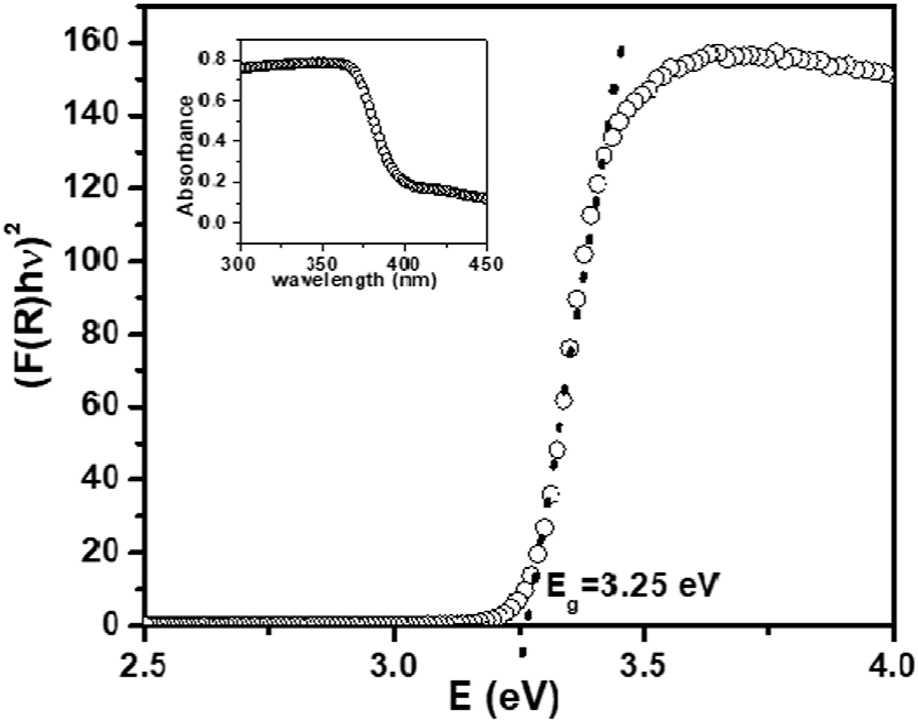
Kubelka- Munk function (KM) versus photon energy for ZnO NPs. Inset- Absorption spectra for synthesized ZnO NPs.

DRS spectra for synthesized NPs showed strong reflection above 365 nm. Absorption spectra for the ZnO NPs synthesized using ethanolic extracts of *E. odoratum* is shown in inset of Figure 4. The absorption peak for synthesized ZnO NPs was observed at 380.5 nm. Researchers have presented reports of both, the red shift due to defect incorporations with extended localized states within the band gap region (Marotti et al., 2006; Kamarulzaman et al., 2015) and the blue shift of E_g_ (Tan et al., 2005; Debanath and Karmakar, 2013).

The bandgap energy of ZnO NPs is calculated using Kubelka-Munk (KM) relation as described in Wetchakun et al. (2012) and Yan et al. (2021).

$$\frac{K}{S}=\frac{{\left(1-R_{\infty }\right)}^{2}}{2R_{\infty }}=F\left(R\right)$$ (2)

Where, F(R) is the remission or Kubelka-Munk (KM) function.

In parabolic band structure,

$$\alpha h\nu =C{\left(h\nu -E_{g}\right)}^{1/2}$$ (3)

where, *α* denotes linear absorption coefficient of the material; *hν* denotes photon energy; C denoted proportionality constant.

For constant scattering coefficient (S) with wavelength, and using Eqs 1, 2,

$${\left[F\left(R\right)h\nu \right]}^{2}=B\left(h\nu -E_{g}\right)$$ (3a)

E_g_ was measured by extrapolating the linear portion of modified KM function and hν, as shown in Figure 4. The optical bandgap for the synthesized NPs was found to be 3.25 eV. The smaller bandgap value as compared to bulk ZnO (∼3.3 eV) may be attributed to defects including, dislocations, stacking faults, zinc and oxygen vacancies, Zn and oxygen interstitial.

### 3.4 Photoluminescence (PL) spectroscopy analysis

**FIGURE 5:**
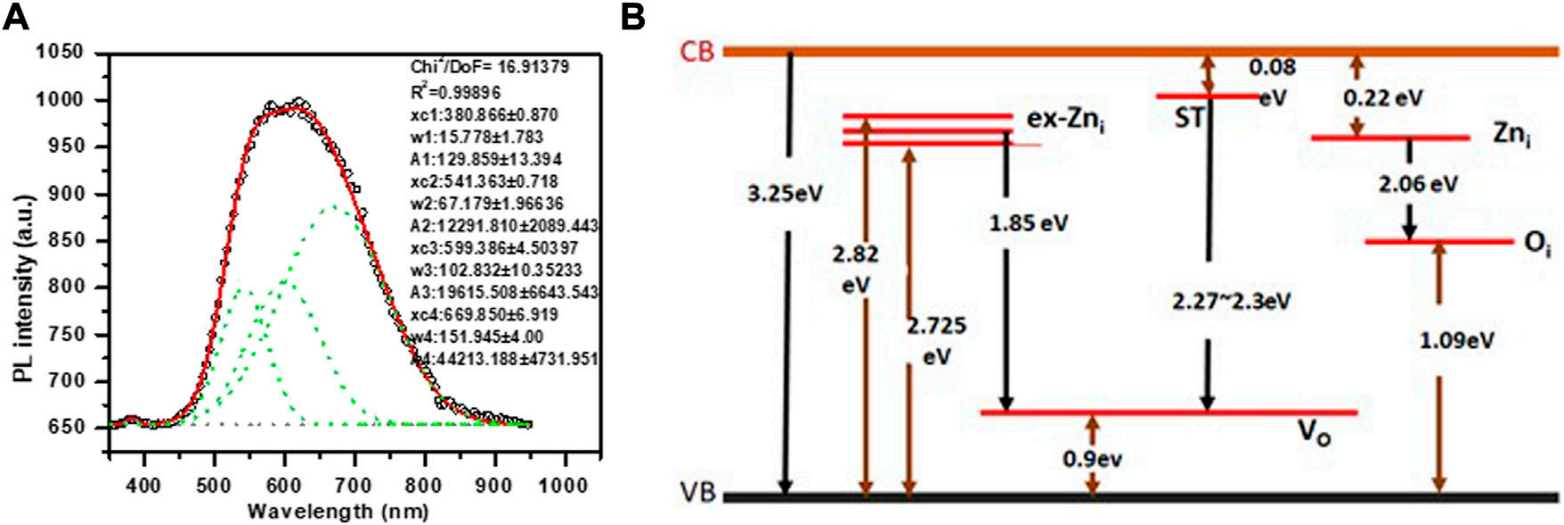
**(A)** Photoluminescence spectra measured at room temperature for green synthesized ZnO NPs. Dotted curve shows the fitted Gaussian peaks to obtain defect states in the ZnO NPs. **(B)** Energy level diagram of synthesized ZnO NPs showing defect states (Vempati et al., 2012) and the possible transition corresponding to observed defect states in PL spectra.

To study the possible defects in the synthesized ZnO NPs, PL spectra was investigated at room temperature. PL spectra of the synthesized sample was recorded using excitation wavelength at 355 nm wavelength with 285 µW power. Emission signal was recorded with CCD (charge Coupled Detector). Optical properties of synthesized ZnO NPs were investigated for its use as coatings in central venous catheter. The nanoparticle size (Choopun et al., 2005; Wang et al., 2005) and surface morphology (Ye et al., 2005; Zhao et al., 2016) as well as defects and synthesis process (Kumar Jangir et al., 2017) affect the PL properties of ZnO. Generally, room temperature PL of ZnO exhibits sharp transition in UV range and a broad transition in visible range. The sharp transition in UV range corresponds to optical transition between electrons in conduction band (CB) and holes in valence band (VB), including excitonic effect (band to band transition). PL originates due to recombination of surface states (Chestnoy et al., 1986). The broad emission is related to dopant/impurities or point defects such as zinc interstitial and oxygen vacancies (Kumar Jangir et al., 2017), etc. Figure 5A shows PL spectra of ZnO nanomaterial. The UV emission at 381.8 nm is attributed to near bandgap excitonic emission (band to band transition). Free exciton in ZnO occur when electron hole pair forms between CB and VB. Second observed peak corresponding to crystalline defects in PL spectra was fitted to Gaussian peak function to obtain corresponding energy level and to access types of defects present in the synthesized sample and their influence on the optical properties.

The number of fitted peaks (Figure 5A) indicate the presence of defect states. The parameters obtained from fitted peaks including peak position, FWHM, area under the curve, are shown in the Figure 5A. Figure 5B shows energy band diagram for the synthesized ZnO NPs. The PL spectra of NPs show intense red emission at 669.85 nm, along with orange and green emission at 599.3 nm and 541.3 nm, respectively. Green emission is attributed transition from surface traps (ST) to oxygen vacancy level (V_O_). Orange emission is due to transition from Zn_i_ level to O_i_ level and red emission is attributed to transition from ex Zn_i_ level to V_O_ level.

### 3.5 Dynamic Light Scattering

**FIGURE 6:**
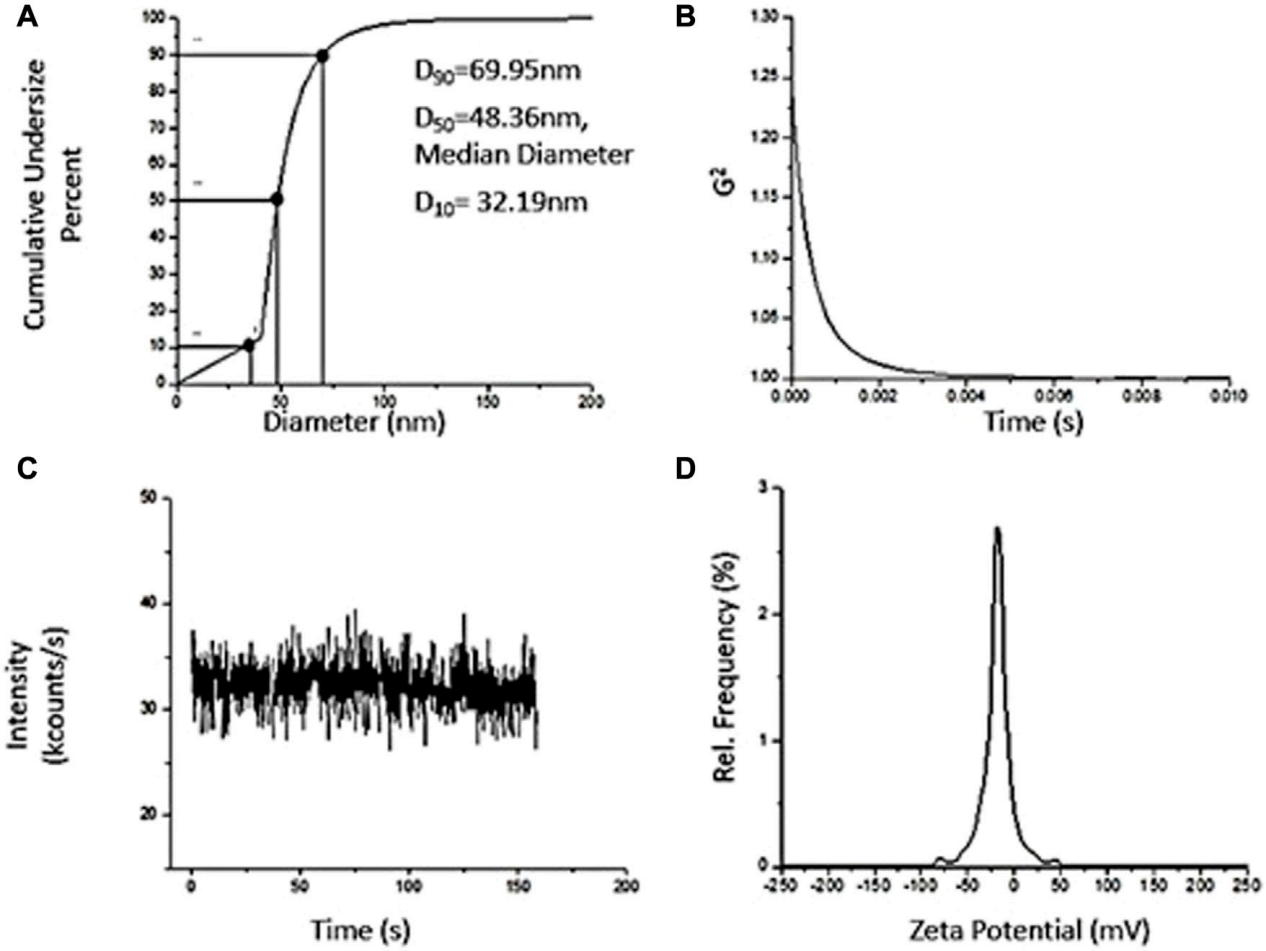
Dynamic Light Scattering Characterization of ZnO synthesized using Green Synthesis. **(A)** Particle Size Distribution, D_10_ = 32.19 nm, Median Diameter, D_50_ = 48.36 nm, D_90_ = 69.95 nm. **(B)** Correlogram. **(C)** Intensity Fluctuation Plot. **(D)** Zeta Potential distribution upon nanoparticles.

The particle size distribution of green synthesized zinc oxide nanoparticles is moderately multimodal with polydispersity index as 0.26 and the hydrodynamic diameter is 142.82 nm. Moreover, d_90_, i.e., 69.95 nm (Figure 6A) represents the size of 90% of particles in the suspension to be below the d_90_ value which is larger than the average particle size, i.e., 34 nm observed in SEM. This is possibly due to the bias of the characterization technique to measure bigger size particles or aggregates (Modena et al., 2019).

The correlogram of ZnO nanoparticles samples decays rapidly (Figure 6B) indicating the composition of NPs suspension by small sized particles, therefore, changing their relative positions rapidly and also, bringing about rapid intensity fluctuations (Figure 6C). The colloid suspensions with ζ-potential in range ∈ (∞, −15] + [15, ∞) are known to be stable (Modena et al., 2019). The colloids are strongly stable if ζ-potential modulus is greater than 25 governed by adequate mutual repulsive forces (Zhou, 2012; Morais et al., 2013). The ζ-potential of ZnO NPs is −15.61 mV (Figure 6D), possessing anionic surface charge and can form a moderately stable colloid. There is a close relationship between the plant extract metabolites and ζ-potential (Lynch et al., 2009). Moreover, the presence of negative charge on NPs due to adsorption of plant extract metabolites reduces aggregation among particles making it a stable dispersion (Vimala et al., 2014).

### 3.6 SEM-EDAX analysis of ZnO NP-coated CVCs

**FIGURE 7:**
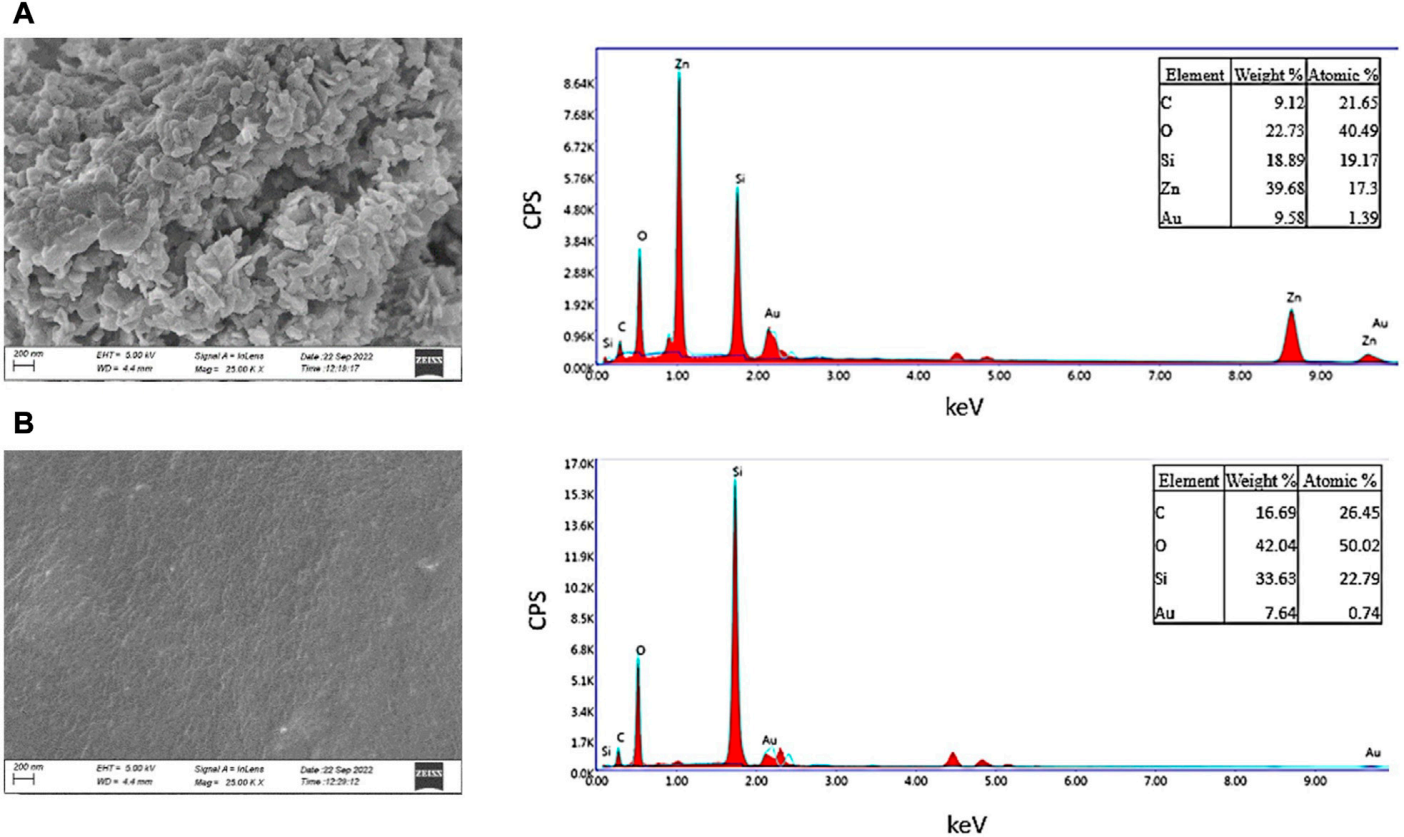
SEM and EDAX Analysis of surface morphology of coated and uncoated catheters. **(A)** Coated Surface of CVC. **(B)** Uncoated Surface of CVC.

The surface of TIVAP showed functionalization of ZnO nanoparticles using SEM-EDAX analysis (Figures 7A, B). The zinc oxide nanoparticles along with HPMC form polymer encapsulated nanoparticles coatings on catheter surfaces as observed in SEM micrograph (Figure 7A). Recently, HPMC based films were developed incorporated with ZnO Nanoparticles for antibacterial wound dressing application (Pitpisutkul and Prachayawarakorn, 2022). Further, more detailed investigations are required to find exact mechanism of chemical interaction between HPMC, ZnO and PDMS surfaces.

### 3.7 ZnO NPs efficiently kill clinically relevant bacteria

**FIGURE 8:**
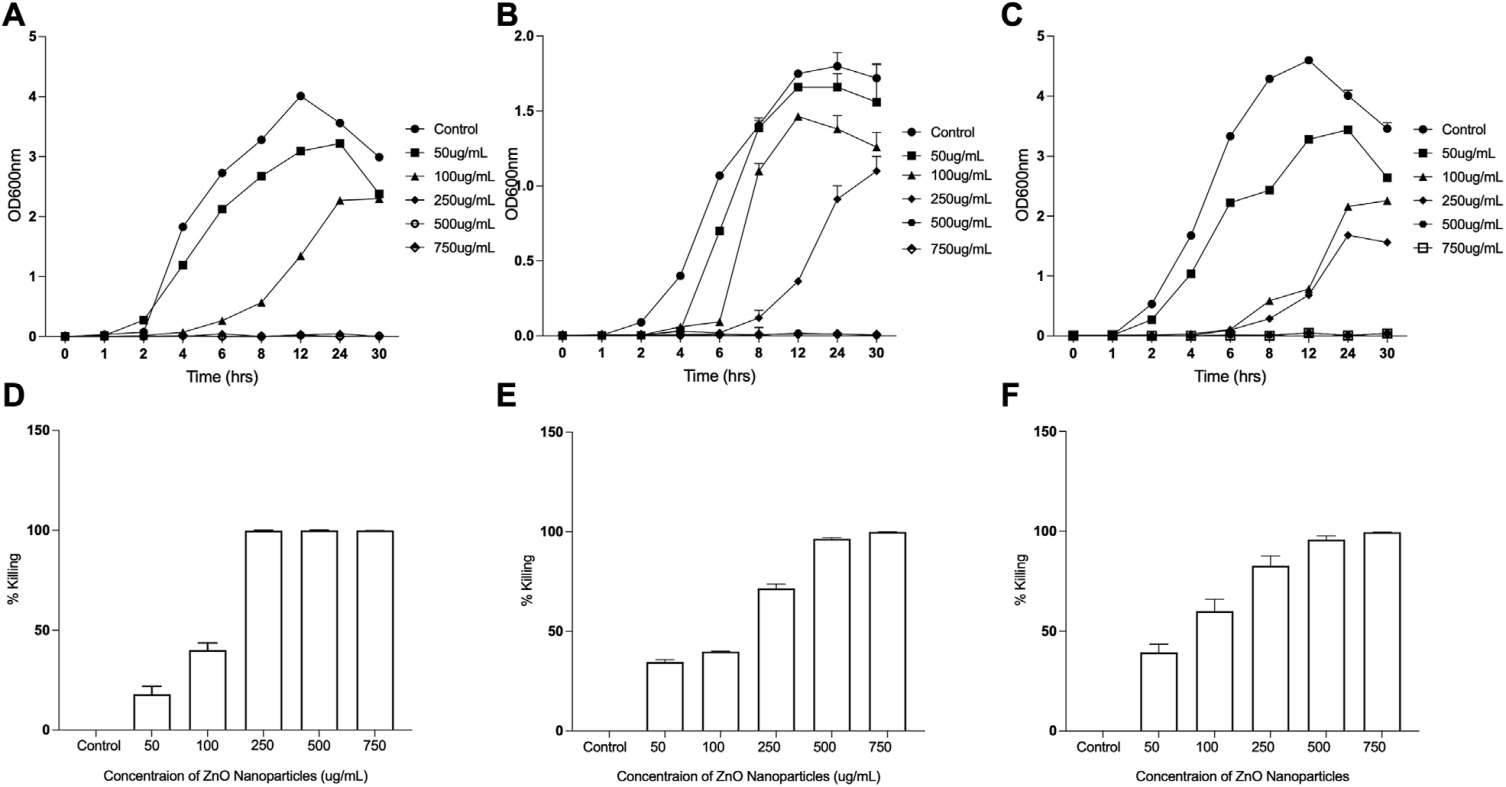
Antibacterial Efficacy of *Eupatorium odoratum* mediated ZnO NPs. Inhibition of: **(A)** *Staphylococcus aureus*, **(B)** *Escherichia coli*, and **(C)** *Proteus aeruginosa* growth in the presence of different concentrations of ZnO NPs; Percentage killing of: **(D)** *Staphylococcus aureus*, **(E)** *Escherichia coli*, and **(F)** *Proteus aeruginosa*, after 24 h of exposure to different concentrations of ZnO NPs.

ZnO exhibits improved antibacterial activity at the nanoscale (Padmavathy and Vijayaraghavan, 2008). The antibacterial properties of ZnO nanoparticles have been reported as a function of its characteristic features. ZnO Nanoparticles when doped with Fe results in significant antibacterial activity against Gram-negative bacteria like *P. aeruginosa*, *E. coli* (Kayani et al., 2018). ZnO nanoparticles consisting flower like morphology (hierarchical structures) make Gram-positive bacteria more susceptible than Gram-negative bacteria (Babayevska et al., 2022). The differential antimicrobial activities of ZnO NPs are due to influence of physical and chemical properties of ZnO NPs obtained by varying synthesis methods, modification of NP surface using doping with metals or capping agent (Gudkov et al., 2021; da Silva et al., 2019). To assess the antibacterial efficacy of ZnO NPs synthesized using ethanolic extracts of *E. odoratum* or chemically synthesized ZnO NPs, exponentially growing bacteria were treated with different concentrations (50 μg/mL to 750 μg/mL) of ZnO NPs for 24 h at 37°C. The viable cell count was estimated by plating the *S. aureus* on TSB agar plates, and *E. coli* and *P. aeruginosa* on LB agar plates. Untreated cultures were used as controls (Figures 8A–F; Supplementary Figure S1). Although a concentration dependent killing was observed upon exposure of all the bacterial strains to either *E. odoratum* mediated or chemically synthesized ZnO NPs, there was no significant difference in the antibacterial activity between the ZnO NPs synthesized by two methods. Although the growth of both Gram-positive as well as Gram-negative strains was inhibited significantly with increasing concentration, growth of *S. aureus* was inhibited up to 99.9% at a concentration of 250 μg/mL *E. odoratum* mediated ZnO NPs. Maximum killing of 99.98% was seen in case of *S. aureus* at 500 μg/mL ZnO NPs whereas 99.9% killing could be achieved only at higher concentration of 750 μg/mL in case of Gram-negative bacteria. This could be due to presence of secondary metabolites (rich in phenols, saponins and tannins) (Inya-agha et al., 1987) in *E. odoratum* extracts, as previously reported, maybe specifically active against Gram-positive bacteria. The literature suggests broad spectrum antibacterial and antifungal activity of *E. odoratum* plant extract. Venkat Raman et al. (Venkata raman et al., 2012) have shown broad spectrum antibacterial activity against Gram-negative and Gram-positive bacteria including MTCC736 *Bacillus subtilis*, MTCC2807 *Corynebacterium glutamicum*, MTCC1572 *E. coli*, MTCC7028 *Klebsiella pneumonia*, MTCC733 *Salmonella typhi*, MTCC87 *S. aureus*, MTCC1938 *Streptococcus thermophilus*, MTCC1771 *P. vulgaris*, MTCC451 *Vibrio parahaemolyticus*. *E. odoratum* extract shows significant (*p* < 0.001) antibacterial activity against all bacterial species excluding *Proteus vulgaris* and *S. typhi*. Besides, *E. odoratum* also show antifungal activity (Ramesh and Subramani, 2018). *Chromolaena odorata* (synonym of *E. odoratum*) extract in combination with antibiotics inhibit growth of *P. aeruginosa* isolated from wound infections. Moreover, *E. odoratum* mediated ZnO NPs are able to kill efficiently both the both the Gram-positive and Gram-negative bacteria, indicating the broad-spectrum activity of these NPs and further warrants the evaluation for probable application in clinical settings. Furthermore, as suggested by other research groups, focused studies are needed to compare the antibacterial efficacies of metal and/or metal oxide NPs using optimized techniques more relevant to nanoparticles (Kourmouli et al., 2018; Masri et al., 2022).

### 3.8 ZnO NPs coating on totally implantable venous access ports (TIVAP) reduce biofilm formation

**FIGURE 9:**
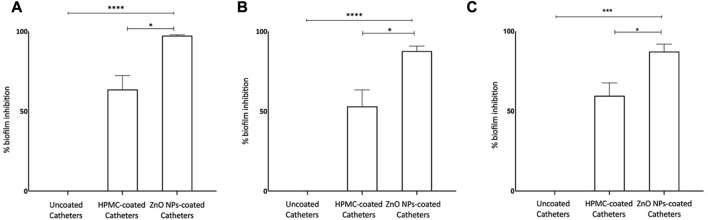
Antibiofilm Efficacy of ZnO NP coated CVCs. **(A)** Biofilm formation of *Staphylococcus aureus* is inhibited by >97%. **(B)** Biofilm formation of *Escherichia coli* is inhibited nearly by 90%. **(C)** Biofilm formation of *Proteus aeruginosa* is inhibited by >90%. ZnO-NPs Coated: ZnO NPs-HPMC composite coating. Statistical analysis was done by 1-way analysis of variance (unpaired *t*-test with Welch’s correction) using GraphPad Prism software (version 8.0.1). Differences were considered significant at *p* < 0.05*, *p* < .0005 ***; *p* < 0.0001 ****.

Zinc oxide nanoparticles are well recognized antibacterial (Liu et al., 2009; El-Masry et al., 2022) and antibiofilm agents, and used to develop antifouling surfaces of medical devices, for example, modifying dental resins (Wang et al., 2019) and denture bases in dental implants (Cierech et al., 2019) and deposition (Wang et al., 2021) and patterning of ZnO NPs on titanium (Ye et al., 2022) for orthopedic implants. We have developed coating of ZnO NPs, synthesized using ethanolic plant extract of *E. odoratum*, on silicone elastomer-based surface of the commercial TIVAP with help of hydroxypropyl methylcellulose (HPMC) as binding agent. ZnO-HPMC based coatings result in formation of hydrophilic surfaces (Rao et al., 2014). The ZnO NPs coated CVCs showed more than 97% inhibition of *S. aureus* biofilm formation and up to 90% inhibition of *E. coli* and *P. aeruginosa* biofilm formation (Figure 9). Hydrophilic surfaces are known to reduce bacterial adhesion and biofilm formation (Lorenzetti et al., 2015; Verhorstert et al., 2020). ZnO NPs based coatings can be tuned to attain hydrophilic surfaces reducing bacterial colonization and inhibiting biofilm formation upon the surfaces (Yong et al., 2015; Kusworo et al., 2021). Our results demonstrated the application of ZnO NPs for the first-time and successful reduction in bacterial formation which can successfully reduce risk of central venous catheter associated infections. Further, *in vivo* studies would validate the antifouling ability in the presence of trilateral interaction between host, device and bacteria.

## 4 Conclusion

HPMC and ZnO are well recognised as safe materials and applied in coatings of food packaging products and wound applications. Zinc oxide nanoparticles were synthesized using plant extract of medicinal plant *E. odoratum* that showed excellent antibacterial activity up to 99.99% killing efficacy. ZnO NPs were coated on commercial TIVAPs using HPMC polymer. The coated CVCs prevented the bacterial biofilm formation of clinically relevant bacteria, viz., *E. coli*, *P. aeruginosa* and *S. aureus* in an *in situ* continuous flow system. Based on our findings, we propose application of green synthesized ZnO NPs (a non-antibiotic based) surface coatings on Central Venous Catheter which has immense potential of improving the patient outcome in clinical settings.

## Data Availability

The original contributions presented in the study are included in the article/Supplementary Material, further inquiries can be directed to the corresponding author.

## Supplementary material

SUPPLEMENTARY FIGURE S1 Comparison of antibacterial efficacy of ZnO NPs synthesized using *E. odoratum* ethanolic extract with ZnO NPs synthesized by chemical method. Inhibition of: **(A)** *S. aureus*, **(B)** *E. coli*, and **(C)** *Proteus aeruginosa* growth in the presence of different concentrations of ZnO NPs.

## References

[B1] Agarwal A. K. Haddad N. J. Vachharajani T. J. Asif A. (2019). Innovations in vascular access for hemodialysis. Kidney Int. 95 (5), 1053–1063. 10.1016/j.kint.2018.11.046 30922666

[B2] Antonelli M. de Pascale G. Ranieri V. M. Pelaia P. Tufano R. Piazza O. (2012). Comparison of triple-lumen central venous catheters impregnated with silver nanoparticles (AgTive®) vs conventional catheters in intensive care unit patients. J. Hosp. Infect. 82 (2), 101–107. 10.1016/j.jhin.2012.07.010 22938728

[B3] Azizi S. Mohamad R. Mahdavi Shahri M. (2017). Green microwave-assisted combustion synthesis of zinc oxide nanoparticles with *Citrullus colocynthis* (L.) schrad: Characterization and biomedical applications. Molecules 22 (2), 301. 10.3390/molecules22020301 28212344 PMC6155814

[B4] Babayevska N. Przysiecka Ł. Iatsunskyi I. Nowaczyk G. Jarek M. Janiszewska E. (2022). ZnO size and shape effect on antibacterial activity and cytotoxicity profile. Sci. Rep. 12 (1), 8148. 10.1038/s41598-022-12134-3 35581357 PMC9114415

[B5] Björling G. Johansson D. Bergström L. Strekalovsky A. Sanchez J. Frostell C. (2018). Evaluation of central venous catheters coated with a noble metal alloy—a randomized clinical pilot study of coating durability, performance and tolerability. J. Biomed. Mater. Res. Part B: Appl. Biomater. 106 (6), 2337–2344. 10.1002/jbm.b.34041 PMC6175140 29106034

[B6] Casimero C. Ruddock T. Hegarty C. Barber R. Devine A. Davis J. (2020). Minimising blood stream infection: Developing new materials for intravascular catheters. Medicines 7 (9), 49. 10.3390/medicines7090049 32858838 PMC7554993

[B7] Chaudhuri S. K. Malodia L. (2017). Biosynthesis of zinc oxide nanoparticles using leaf extract of calotropis gigantea: Characterization and its evaluation on tree seedling growth in nursery stage. Appl. Nanosci. 7 (8), 501–512. 10.1007/s13204-017-0586-7

[B8] Chauhan A. Lebeaux D. Decante B. Kriegel I. Escande M.-C. Ghigo J.-M. (2012). A rat model of central venous catheter to study establishment of long-term bacterial biofilm and related acute and chronic infections. PLOS ONE 7 (5), e37281. 10.1371/journal.pone.0037281 22615964 PMC3353920

[B9] Chauhan A. Bernardin A. Mussard W. Kriegel I. Estève M. Ghigo J.-M. (2014). Preventing Biofilm Formation and associated occlusion by biomimetic glycocalyxlike polymer in central venous catheters. J. Infect. Dis. 210 (9), 1347–1356. 10.1093/infdis/jiu249 24795479

[B10] Chestnoy N. Harris T. D. Hull R. Brus L. E. (1986). Luminescence and photophysics of cadmium sulfide semiconductor clusters: The nature of the emitting electronic state. J. Phys. Chem. 90 (15), 3393–3399. 10.1021/j100406a018

[B11] Choopun S. Hongsith N. Tanunchai S. Chairuangsri T. Krua-in C. Singkarat S. (2005). Single-crystalline ZnO nanobelts by RF sputtering. J. Cryst. Growth 282 (3), 365–369. 10.1016/j.jcrysgro.2005.05.020

[B12] Chopra V. O’Horo J. C. Rogers M. A. M. Maki D. G. Safdar N. (2013). The risk of bloodstream infection associated with peripherally inserted central catheters compared with central venous catheters in adults: A systematic review and meta-analysis. Infect. Control Hosp. Epidemiol. 34 (9), 908–918. 10.1086/671737 23917904

[B13] Cierech M. Wojnarowicz J. Kolenda A. Krawczyk-Balska A. Prochwicz E. Woźniak B. (2019). Zinc oxide nanoparticles cytotoxicity and release from newly formed PMMA–ZnO nanocomposites designed for denture bases. Nanomaterials 9 (9), 1318. 10.3390/nano9091318 31540147 PMC6781076

[B14] Cinteză L. O. Tănase M. A. (2020). “Multifunctional ZnO nanoparticle: Based coatings for cultural heritage preventive conservation,” in Thin films. Editor Ares A. E. (IntechOpen). Ch. 6. 10.5772/intechopen.94070

[B15] Copetti R. de Monte A. (2005). Rapid echocardiographic diagnosis of suspected paradoxical gas embolism after central venous catheter placement in the upright position. Echocardiography 22 (8), 695–696. 10.1111/j.1540-8175.2005.40082.x 16174129

[B16] Corral L. Nolla-Salas M. Ibañez-Nolla J. León M. A. Díaz R. M. Cruz Martín M. (2003). A prospective, randomized study in critically ill patients using the Oligon Vantex® catheter. J. Hosp. Infect. 55 (3), 212–219. 10.1016/j.jhin.2003.07.001 14572489

[B17] da Silva B. L. Paiva Abuçafy M. Berbel Manaia E. Augusto Oshiro Junior J. Chiari-Andréo B. G. Cl R Pietro R. (2019). Relationship between structure and antimicrobial activity of zinc oxide nanoparticles: An overview. Int. J. Nanomed. 14 (14), 9395–9410. 10.2147/IJN.S216204 PMC6897062 31819439

[B18] David L. Jean-Marc G. Christophe B. (2014). Biofilm-related infections: Bridging the gap between clinical management and fundamental aspects of recalcitrance toward antibiotics. Microbiol. Mol. Biol. Rev. 78 (3), 510–543. 10.1128/MMBR.00013-14 25184564 PMC4187679

[B19] Debanath M. K. Karmakar S. (2013). Study of blueshift of optical band gap in zinc oxide (ZnO) nanoparticles prepared by low-temperature wet chemical method. Mater. Lett. 111, 116–119. 10.1016/j.matlet.2013.08.069

[B20] Debbarma M. Pala N. A. Kumar M. Bussmann R. W. (2017). Traditional knowledge of medicinal plants in tribes of Tripura in northeast India. Afr. J. Tradit. Complement. Altern. Med. 14 (4), 156–168. 10.21010/ajtcam.v14i4.19 28638879 PMC5471463

[B21] Doan Thi T. U. Nguyen T. T. Thi Y. D. Ta Thi K. H. Phan B. T. Pham K. N. (2020). Green synthesis of ZnO nanoparticles using orange fruit peel extract for antibacterial activities. RSC Adv. 10 (40), 23899–23907. 10.1039/D0RA04926C 35517333 PMC9055061

[B22] El-Masry R. M. Talat D. Hassoubah S. A. Zabermawi N. M. Eleiwa N. Z. Sherif R. M. (2022). Evaluation of the antimicrobial activity of ZnO nanoparticles against enterotoxigenic *Staphylococcus aureus* . Life 12 (10), 1662. 10.3390/life12101662 36295097 PMC9605543

[B23] Faisal S. Jan H. Shah S. A. Shah S. Khan A. Akbar M. T. (2021). Green synthesis of zinc oxide (ZnO) nanoparticles using aqueous fruit extracts of myristica fragrans: Their characterizations and biological and environmental applications. ACS Omega 6 (14), 9709–9722. 10.1021/acsomega.1c00310 33869951 PMC8047667

[B24] Ferreira Chacon J. M. Hato de Almeida E. de Lourdes Simões R. Lazzarin C. Ozório V. Alves B. C. (2011). Randomized study of minocycline and edetic acid as a locking solution for central line (Port-A-Cath) in children with cancer. Chemotherapy 57 (4), 285–291. 10.1159/000328976 21778716

[B25] Gudkov S. v. Burmistrov D. E. Serov D. A. Rebezov M. B. Semenova A. A. Lisitsyn A. B. (2021). A mini review of antibacterial properties of ZnO nanoparticles. Front. Phys. 9. 10.3389/fphy.2021.641481 PMC8300809 34356805

[B26] Guleri A. Kumar A. Morgan R. J. M. Hartley M. Roberts D. H. (2012). Anaphylaxis to chlorhexidine-coated central venous catheters: A case series and review of the literature. Surg. Infect. 13 (3), 171–174. 10.1089/sur.2011.011 22568873

[B27] Hanna H. Benjamin R. Chatzinikolaou I. Alakech B. Richardson D. Mansfield P. (2004). Long-term silicone central venous catheters impregnated with minocycline and rifampin decrease rates of catheter-related bloodstream infection in cancer patients: A prospective randomized clinical trial. J. Clin. Oncol. 22 (15), 3163–3171. 10.1200/JCO.2004.04.124 15284269

[B28] Husain F. M. Zia Q. Khan M. F. Ahmad E. Jamal A. Alaidarous M. (2020). Anti-quorum sensing and anti-biofilm activity of zinc oxide nanospikes. ACS Omega 5 (50), 32203–32215. 10.1021/acsomega.0c03634 33376858 PMC7758897

[B29] Huygh J. Peeters Y. Bernards J. Malbrain M. (2016). Hemodynamic monitoring in the critically ill: An overview of current cardiac output monitoring methods [version 1; peer review: 3 approved]. F1000Research 5, 2855. 10.12688/f1000research.8991.1 PMC5166586 28003877

[B30] Inya-agha S. I. Oguntimein B. O. Sofowora A. Benjamin T. v. (1987). Phytochemical and antibacterial studies on the essential oil of eupatorium odoratum. Int. J. Crude Drug Res. 25 (1), 49–52. 10.3109/13880208709060911

[B31] Iqbal J. Abbasi B. A. Yaseen T. Zahra S. A. Shahbaz A. Shah S. A. (2021). Green synthesis of zinc oxide nanoparticles using Elaeagnus angustifolia L. leaf extracts and their multiple *in vitro* biological applications. Sci. Rep. 11 (1), 20988. 10.1038/s41598-021-99839-z 34697404 PMC8545962

[B32] Ivanova A. Ivanova K. Perelshtein I. Gedanken A. Todorova K. Milcheva R. (2021). Sonochemically engineered nano-enabled zinc oxide/amylase coatings prevent the occurrence of catheter-associated urinary tract infections. Mater. Sci. Eng. C 131, 112518. 10.1016/j.msec.2021.112518 34857297

[B102] Jeffery G. A. (1957). “Elements of x-ray diffraction,” in Journal of Chemical Education. Editors Cullity B. D. 34 (4), A178. 10.1021/ed034pA178

[B33] Kamarulzaman N. Kasim M. F. Rusdi R. (2015). Band gap narrowing and widening of ZnO nanostructures and doped materials. Nanoscale Res. Lett. 10 (1), 346. 10.1186/s11671-015-1034-9 26319225 PMC4552709

[B34] Kayani Z. N. Abbas E. Saddiqe Z. Riaz S. Naseem S. (2018). Photocatalytic, antibacterial, optical and magnetic properties of Fe-doped ZnO nano-particles prepared by sol-gel. Mater. Sci. Semicond. Process. 88, 109–119. 10.1016/j.mssp.2018.08.003

[B35] Kim H. J. Yun J. Kim H. J. Kim K. H. Kim S. H. Lee S.-C. (2010). Safety and effectiveness of central venous catheterization in patients with cancer: Prospective observational study. J. Korean Med. Sci. 25 (12), 1748–1753. 10.3346/jkms.2010.25.12.1748 21165289 PMC2995228

[B36] Kourmouli A. Valenti M. van Rijn E. Beaumont H. J. E. Kalantzi O.-I. Schmidt-Ott A. (2018). Can disc diffusion susceptibility tests assess the antimicrobial activity of engineered nanoparticles? J. Nanopart. Res. 20 (3), 62. 10.1007/s11051-018-4152-3 29527123 PMC5834581

[B37] Krikava I. Kolar M. Garajova B. Balik T. Sevcikova A. Pachl J. (2011). Polyhexanide anti-infective coating of central venous catheters in prevention of catheter colonization and bloodstream infection: Study HC-G-H-0507. Crit. Care 15 (1), P229. 10.1186/cc9649

[B38] Kumar Jangir L. Kumari Y. Kumar A. Kumar M. Awasthi K. (2017). Investigation of luminescence and structural properties of ZnO nanoparticles, synthesized with different precursors. Mater. Chem. Front. 1 (7), 1413–1421. 10.1039/C7QM00058H

[B39] Kusworo T. D. Dalanta F. Aryanti N. Othman N. H. (2021). Intensifying separation and antifouling performance of PSf membrane incorporated by GO and ZnO nanoparticles for petroleum refinery wastewater treatment. J. Water Process Eng. 41, 102030. 10.1016/j.jwpe.2021.102030

[B40] LaBella G. D. Tang J. (2012). “Removal of totally implantable venous access device,” in Totally implantable venous access devices: Management in mid- and long-term clinical setting. Editors Niederhuber J. E. di Carlo I. Biffi R. (Springer Milan), 247–255. 10.1007/978-88-470-2373-4_35

[B41] Lebeaux D. Chauhan A. Létoffé S. Fischer F. de Reuse H. Beloin C. (2014a). pH-mediated potentiation of aminoglycosides kills bacterial persisters and eradicates *in vivo* biofilms. J. Infect. Dis. 210 (9), 1357–1366. 10.1093/infdis/jiu286 24837402

[B42] Lebeaux D. Fernández-Hidalgo N. Chauhan A. Lee S. Ghigo J.-M. Almirante B. (2014b). Management of infections related to totally implantable venous-access ports: Challenges and perspectives. Lancet. Infect. Dis. 14 (2), 146–159. 10.1016/S1473-3099(13)70266-4 24314751

[B43] Liu Y. He L. Mustapha A. Li H. Hu Z. Q. Lin M. (2009). Antibacterial activities of zinc oxide nanoparticles against *Escherichia coli* O157:H7. J. Appl. Microbiol. 107 (4), 1193–1201. 10.1111/j.1365-2672.2009.04303.x 19486396

[B44] Lorenzetti M. Dogša I. Stošicki T. Stopar D. Kalin M. Kobe S. (2015). The influence of surface modification on bacterial adhesion to titanium-based substrates. ACS Appl. Mater. Interfaces 7 (3), 1644–1651. 10.1021/am507148n 25543452

[B45] Luković Golić D. Branković G. Počuča Nešić M. Vojisavljević K. Rečnik A. Daneu N. (2011). Structural characterization of self-assembled ZnO nanoparticles obtained by the sol–gel method from Zn(CH3COO)2·2H2O. Nanotechnology 22 (39), 395603. 10.1088/0957-4484/22/39/395603 21891855

[B46] Lynch I. Salvati A. Dawson K. A. (2009). What does the cell see? Nat. Nanotechnol. 4 (9), 546–547. 10.1038/nnano.2009.248 19734922

[B47] Mahamuni-Badiger P. P. Patil P. M. Badiger M. v. Patel P. R. Thorat-Gadgil B. S. Pandit A. (2020). “Biofilm formation to inhibition: Role of zinc oxide-based nanoparticles,” in Materials science and engineering C (Elsevier), 108. 10.1016/j.msec.2019.110319 31923962

[B48] Marco R. Laura M. Olga R.-N. Celia C. Pedro P.-A. Marta H.-M. (2018). Short-term peripheral venous catheter-related bloodstream infections: Evidence for increasing prevalence of gram-negative microorganisms from a 25-year prospective observational study. Antimicrob. Agents Chemother. 62 (11), 008922–e918. 10.1128/AAC.00892-18 PMC6201066 30126952

[B49] Marotti R. E. Giorgi P. Machado G. Dalchiele E. A. (2006). Crystallite size dependence of band gap energy for electrodeposited ZnO grown at different temperatures. Sol. Energy Mater. Sol. Cells 90 (15), 2356–2361. 10.1016/j.solmat.2006.03.008

[B50] Masri A. Brown D. M. Smith D. G. E. Stone V. Johnston H. J. (2022). Comparison of *in vitro* approaches to assess the antibacterial effects of nanomaterials. J. Funct. Biomater. 13 (4), 255. 10.3390/jfb13040255 36412895 PMC9703965

[B51] Mathur P. Malpiedi P. Walia K. Srikantiah P. Gupta S. Lohiya A. (2022). Health-care-associated bloodstream and urinary tract infections in a network of hospitals in India: A multicentre, hospital-based, prospective surveillance study. Lancet Glob. Health 10 (9), e1317–e1325. 10.1016/S2214-109X(22)00274-1 35961355

[B52] Memarzadeh K. Sharili A. S. Huang J. Rawlinson S. C. F. Allaker R. P. (2015). Nanoparticulate zinc oxide as a coating material for orthopedic and dental implants. J. Biomed. Mater. Res. Part A 103 (3), 981–989. 10.1002/jbm.a.35241 24862288

[B53] Mermel L. A. Allon M. Bouza E. Craven D. E. Flynn P. O’Grady N. P. (2009). Clinical practice guidelines for the diagnosis and management of intravascular catheter-related infection: 2009 update by the infectious diseases society of America. Clin. Infect. Dis. 49 (1), 1–45. 10.1086/599376 19489710 PMC4039170

[B54] Modena M. M. Rühle B. Burg T. P. Wuttke S. (2019). Nanoparticle characterization: What to measure? Adv. Mater. 31 (32), 1901556. 10.1002/adma.201901556 31148285

[B55] Moradpoor H. Safaei M. Mozaffari H. R. Sharifi R. Imani M. M. Golshah A. (2021). An overview of recent progress in dental applications of zinc oxide nanoparticles. RSC Adv. 11 (34), 21189–21206. 10.1039/D0RA10789A 35479373 PMC9034121

[B56] Morais J. P. S. Rosa M. D. F. de Souza Filho M. de sá M. Nascimento L. D. do Nascimento D. M. (2013). Extraction and characterization of nanocellulose structures from raw cotton linter. Carbohydr. Polym. 91 (1), 229–235. 10.1016/j.carbpol.2012.08.010 23044127

[B57] Naseer M. Aslam U. Khalid B. Chen B. (2020). Green route to synthesize Zinc Oxide Nanoparticles using leaf extracts of Cassia fistula and Melia azadarach and their antibacterial potential. Sci. Rep. 10 (1), 9055. 10.1038/s41598-020-65949-3 32493935 PMC7270115

[B58] Niyyar V. D. (2012). Catheter dysfunction: The role of lock solutions. Semin. Dial. 25 (6), 693–699. 10.1111/j.1525-139X.2011.00991.x 22175421

[B59] O’Grady N. P. Alexander M. Burns L. A. Dellinger E. P. Garland J. Heard S. O. (2011). Guidelines for the prevention of intravascular catheter-related infections. Clin. Infect. Dis. 52 (9), e162–e193. 10.1093/cid/cir257 21460264 PMC3106269

[B60] Ogunyemi S. O. Abdallah Y. Zhang M. Fouad H. Hong X. Ibrahim E. (2019). Green synthesis of zinc oxide nanoparticles using different plant extracts and their antibacterial activity against Xanthomonas oryzae pv. oryzae. Artif. Cells, Nanomed. Biotechnol. 47 (1), 341–352. 10.1080/21691401.2018.1557671 30691311

[B61] Padmavathy N. Vijayaraghavan R. (2008). Enhanced bioactivity of ZnO nanoparticles—An antimicrobial study. Sci. Technol. Adv. Mater. 9 (3), 035004. 10.1088/1468-6996/9/3/035004 27878001 PMC5099658

[B62] Pitpisutkul V. Prachayawarakorn J. (2022). Hydroxypropyl methylcellulose/carboxymethyl starch/zinc oxide porous nanocomposite films for wound dressing application. Carbohydr. Polym. 298, 120082. 10.1016/j.carbpol.2022.120082 36241320

[B63] Pittiruti M. Hamilton H. Biffi R. MacFie J. Pertkiewicz M. (2009). ESPEN guidelines on parenteral nutrition: Central venous catheters (access, care, diagnosis and therapy of complications). Clin. Nutr. 28(4), 365–377. 10.1016/j.clnu.2009.03.015 19464090

[B64] Prasad K. S. Prasad S. K. Veerapur R. Lamraoui G. Prasad A. Prasad M. N. N. (2021). Antitumor potential of green synthesized ZnONPs using root extract of Withania somnifera against human breast cancer cell line. Separations 8 (1), 8. 10.3390/separations8010008

[B65] Priyadharshini S. S. Shubha J. P. Shivalingappa J. Adil S. F. Kuniyil M. Hatshan M. R. (2022). Photocatalytic degradation of methylene blue and metanil yellow dyes using green synthesized zinc oxide (ZnO) nanocrystals. Crystals 12 (1), 22. 10.3390/cryst12010022

[B66] Puspasari V. Ridhova A. Hermawan A. Amal M. I. Khan M. M. (2022). ZnO-based antimicrobial coatings for biomedical applications. Bioprocess Biosyst. Eng. 45 (9), 1421–1445. 10.1007/s00449-022-02733-9 35608710 PMC9127292

[B67] Qing Y. Yang C. Hu C. Zheng Y. Liu C. (2015). A facile method to prepare superhydrophobic fluorinated polysiloxane/ZnO nanocomposite coatings with corrosion resistance. Appl. Surf. Sci. 326, 48–54. 10.1016/j.apsusc.2014.11.100

[B68] Raad I. Darouiche R. Hachem R. Mansouri M. Bodey G. P. (1996). The broad-spectrum activity and efficacy of catheters coated with minocycline and rifampin. J. Infect. Dis. 173 (2), 418–424. 10.1093/infdis/173.2.418 8568304

[B69] Ramesh P. Subramani A. (2018). Effect of antimicrobial activity of Eupatorium odoratum against clinical microbes. Int. J. Sci. Res. Biol. Sci. 5 (5), 30–35. 10.26438/ijsrbs/v5i5.3035

[B70] Ranpariya B. Salunke G. Karmakar S. Babiya K. Sutar S. Kadoo N. (2021). Antimicrobial synergy of silver-platinum nanohybrids with antibiotics. Front. Microbiol. 11, 610968. 10.3389/fmicb.2020.610968 33597929 PMC7882503

[B71] Rao B. L. Asha S. Madhukumar R. Latha S. Gowda M. Shetty G. R. (2014). Structural, surface wettability and antibacterial properties of HPMC-ZnO nanocomposite. AIP Conf. Proc. 1591 (1), 807–809. 10.1063/1.4872763

[B72] Ricardo S. I. C. Anjos I. I. L. Monge N. Faustino C. M. C. Ribeiro I. A. C. (2020). A glance at antimicrobial strategies to prevent catheter-associated medical infections. ACS Infect. Dis. 6 (12), 3109–3130. 10.1021/acsinfecdis.0c00526 33245664

[B73] Safdar N. Maki D. G. (2006). Use of vancomycin-containing lock or flush solutions for prevention of bloodstream infection associated with central venous access devices: A meta-analysis of prospective, randomized trials. Clin. Infect. Dis. 43 (4), 474–484. 10.1086/505976 16838237

[B74] Sampath L. A. Tambe S. M. Modak S. M. (2001). *In vitro* and *in vivo* efficacy of catheters impregnated with antiseptics or antibiotics: Evaluation of the risk of bacterial resistance to the antimicrobials in the catheters. Infect. Control Hosp. Epidemiol. 22 (10), 640–646. 10.1086/501836 11776351

[B75] Saravanan M. Gopinath V. Chaurasia M. K. Syed A. Ameen F. Purushothaman N. (2018). Green synthesis of anisotropic zinc oxide nanoparticles with antibacterial and cytofriendly properties. Microb. Pathog. 115, 57–63. 10.1016/j.micpath.2017.12.039 29248514

[B76] Shah H. Bosch W. Thompson K. M. Hellinger W. C. (2013). Intravascular catheter-related bloodstream infection. Neurohospitalist 3 (3), 144–151. 10.1177/1941874413476043 24167648 PMC3805442

[B77] Singh B. P. Ghosh S. Chauhan A. (2021). Development, dynamics and control of antimicrobial-resistant bacterial biofilms: A review. Environ. Chem. Lett. 19 (3), 1983–1993. 10.1007/s10311-020-01169-5

[B78] Smith R. N. Nolan J. P. (2013). Central venous catheters. Br. Med. J. 347, f6570. 10.1136/bmj.f6570 24217269

[B79] Stabryla L. M. Johnston K. A. Diemler N. A. Cooper V. S. Millstone J. E. Haig S.-J. (2021). Role of bacterial motility in differential resistance mechanisms of silver nanoparticles and silver ions. Nat. Nanotechnol. 16 (9), 996–1003. 10.1038/s41565-021-00929-w 34155383

[B80] Stressmann Franziska A. Elodie C.-D. Delphine C. Ashwini C. Aimee W. Sylvaine D.-F. (2017). Comparative analysis of bacterial community composition and structure in clinically symptomatic and asymptomatic central venous catheters. MSphere 2 (5), 001466–e217. 10.1128/mSphere.00146-17 PMC5615130 28959736

[B81] Tan S. T. Chen B. Sun X. Fan W. Kwok H. Zhang X. (2005). Blueshift of optical band gap in ZnO thin films grown by metal-organic chemical-vapor deposition. J. Appl. Phys. 98, 013505. 10.1063/1.1940137

[B82] Tokumoto M. S. Pulcinelli S. H. Santilli C. Briois V. (2002). Catalysis and temperature dependence on the formation of ZnO nanoparticles and of zinc acetate derivatives prepared by the Sol−Gel route. J. Phys. Chem. B 107, 568–574. 10.1021/jp0217381

[B83] Vempati S. Mitra J. Dawson P. (2012). One-step synthesis of ZnO nanosheets: A blue-white fluorophore. Nanoscale Res. Lett. 7 (1), 470. 10.1186/1556-276X-7-470 22908931 PMC3522028

[B84] Venkata raman B. La S. Pardha Saradhi M. Narashimha Rao B. Naga Vamsi Krishna A. Radhakrishnan T. M. (2012). Antibacterial, antioxidant activity and GC-MS analysis of Eupatorium odoratum. Asian J. Pharm. Clin. Res. 5.

[B85] Verbič A. Gorjanc M. Simončič B. (2019). Zinc oxide for functional textile coatings: Recent advances. Coatings 9 (9), 550. 10.3390/coatings9090550

[B86] Verhorstert K. W. J. Guler Z. de Boer L. Riool M. Roovers J.-P. W. R. Zaat S. A. J. (2020). *In vitro* bacterial adhesion and Biofilm Formation on fully absorbable poly-4-hydroxybutyrate and nonabsorbable polypropylene pelvic floor implants. ACS Appl. Mater. Interfaces 12 (48), 53646–53653. 10.1021/acsami.0c14668 33210919 PMC7716345

[B87] Vimala K. Sundarraj S. Paulpandi M. Vengatesan S. Kannan S. (2014). Green synthesized doxorubicin loaded zinc oxide nanoparticles regulates the Bax and Bcl-2 expression in breast and colon carcinoma. Process Biochem., 49 (1), 160–172. 10.1016/j.procbio.2013.10.007

[B88] Walder B. Pittet D. Tramèr M. R. (2002). Prevention of bloodstream infections with central venous catheters treated with anti-infective agents depends on catheter type and insertion time: Evidence from a meta-analysis. Infect. Control Hosp. Epidemiol. 23 (12), 748–756. 10.1086/502005 12517018

[B89] Wang R. Liu C.-P. Huang J. Chen S.-J. (2005). ZnO symmetric nanosheets integrated with nanowalls. Appl. Phys. Lett. 87, 053103. 10.1063/1.2005386

[B90] Wang H. Tong H. Liu H. Wang Y. Wang R. Gao H. (2018). Effectiveness of antimicrobial-coated central venous catheters for preventing catheter-related blood-stream infections with the implementation of bundles: A systematic review and network meta-analysis. Ann. Intensive Care 8 (1), 71. 10.1186/s13613-018-0416-4 29904809 PMC6002334

[B91] Wang Y. Hua H. Li W. Wang R. Jiang X. Zhu M. (2019). Strong antibacterial dental resin composites containing cellulose nanocrystal/zinc oxide nanohybrids. J. Dent. 80, 23–29. 10.1016/j.jdent.2018.11.002 30423354

[B92] Wang Z. Wang X. Wang Y. Zhu Y. Liu X. Zhou Q. (2021). NanoZnO-modified titanium implants for enhanced anti-bacterial activity, osteogenesis and corrosion resistance. J. Nanobiotechnol. 19 (1), 353. 10.1186/s12951-021-01099-6 PMC8557588 34717648

[B93] Wetchakun N. Chaiwichain S. Inceesungvorn B. Pingmuang K. Phanichphant S. Minett A. I. (2012). BiVO4/CeO2 nanocomposites with high visible-light-induced photocatalytic activity. ACS Appl. Mater. Interfaces 4 (7), 3718–3723. 10.1021/am300812n 22746549

[B94] Wolf J. Shenep J. L. Clifford V. Curtis N. Flynn P. M. (2013). Ethanol lock therapy in pediatric hematology and oncology. Pediatr. Blood Cancer 60 (1), 18–25. 10.1002/pbc.24249 22911535

[B95] Wu E. (1989). POWD, an interactive program for powder diffraction data interpretation and indexing. J. Appl. Crystallogr. 22 (5), 506–510. 10.1107/S0021889889005066

[B96] Yan X. Xiao X. Au C. Mathur S. Huang L. Wang Y. (2021). Electrospinning nanofibers and nanomembranes for oil/water separation. J. Mater. Chem. A 9 (38), 21659–21684. 10.1039/D1TA05873H

[B97] Ye J. D. Gu S. L. Qin F. Zhu S. M. Liu S. M. Zhou X. (2005). Correlation between green luminescence and morphology evolution of ZnO films. Appl. Phys. A 81 (4), 759–762. 10.1007/s00339-004-2996-0

[B98] Ye J. Li B. Li M. Zheng Y. Wu S. Han Y. (2022). Formation of a ZnO nanorods-patterned coating with strong bactericidal capability and quantitative evaluation of the contribution of nanorods-derived puncture and ROS-derived killing. Bioact. Mater. 11, 181–191. 10.1016/j.bioactmat.2021.09.019 34938922 PMC8665260

[B99] Yong H. E. Krishnamoorthy K. Hyun K. T. Kim S. J. (2015). Preparation of ZnO nanopaint for marine antifouling applications. J. Indust. Eng. Chem. 29, 39–42. 10.1016/j.jiec.2015.04.020

[B100] Zhao J.-H. Liu C.-J. Lv Z.-H. (2016). Photoluminescence of ZnO nanoparticles and nanorods. Optik 127 (3), 1421–1423. 10.1016/j.ijleo.2015.11.018

[B101] Zhou Y. Fu S. Y. Zheng L. M. Zhan H. Y. (2012). Effect of nanocellulose isolation techniques on the formation of reinforced poly(vinyl alcohol) nanocomposite films. Express Polym. Lett. 6, 794–804. 10.3144/expresspolymlett.2012.85

