# Supplementary figures and images for: Phyto-assisted synthesis of zinc oxide nanoparticles for developing antibiofilm surface coatings on central venous catheters
Source: Front Chem. 2023 Mar 23;11:1138333. doi: 10.3389/fchem.2023.1138333 (PMC10076889; doi:10.3389/fchem.2023.1138333)

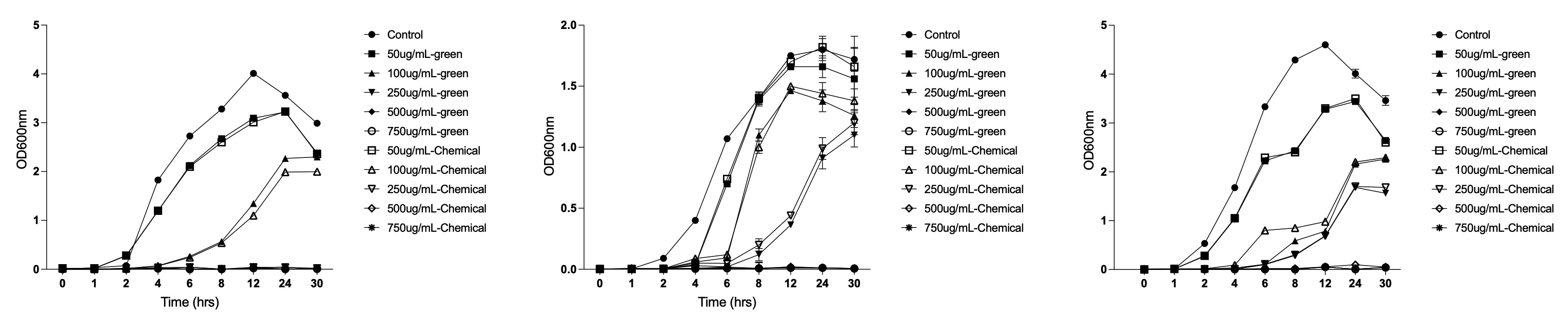

Supplement: Supplementary file 1 [file Image1.jpeg]
