# Supplementary material for: Phyto-assisted synthesis of zinc oxide nanoparticles for developing antibiofilm surface coatings on central venous catheters
Source: Front Chem. 2023 Mar 23;11:1138333. doi: 10.3389/fchem.2023.1138333 (PMC10076889; doi:10.3389/fchem.2023.1138333)
Supplement: Supplementary file 2 [file DataSheet1.docx]

**Supplementary Information**

Comparison of antibacterial efficacy of ZnO NPs synthesized using *E. odoratum* ethanolic extract with ZnO NPs synthesized by chemical method (Supplementary Figure 1).
